# A Highly Conserved Epitope (RNNQIPQDF) of *Porcine teschovirus* Induced a Group-Specific Antiserum: A Bioinformatics-Predicted Model with Pan-PTV Potential

**DOI:** 10.3390/v12111225

**Published:** 2020-10-29

**Authors:** Tung-Hsuan Tsai, Chia-Yi Chang, Fun-In Wang

**Affiliations:** 1 School of Veterinary Medicine, National Taiwan University, No. 1, Section 4, Roosevelt Road, Taipei 10617, Taiwan; d99629004@ntu.edu.tw; 2 OIE Reference Expert for CSF, Animal Health Research Institute, Council of Agriculture, Executive Yuan, No. 376, Chung Cheng Road, Tansui, New Taipei City 25158, Taiwan

**Keywords:** bioinformatics, linearly arranged epitope, *Porcine teschovirus*

## Abstract

*Porcine teschovirus* (PTV) is an OIE-listed pathogen with 13 known PTV serotypes. Heterologous PTV serotypes frequently co-circulate and co-infect with another swine pathogen, causing various symptoms in all age groups, thus highlighting the need for a pan-PTV diagnostic tool. Here, a recombinant protein composed of a highly conserved “RNNQIPQDF” epitope on the GH loop of VP1, predicted in silico, and a tandem repeat of this epitope carrying the pan DR (PADRE) and Toxin B epitopes was constructed to serve as a PTV detection tool. This recombinant GST-PADRE-(RNNQIPQDF)^n^-Toxin B protein was used as an immunogen, which effectively raised non-neutralizing or undetectable neutralizing antibodies against PTV in mice. The raised antiserum was reactive against all the PTV serotypes (PTV–1–7) tested, but not against members of the closely related genera *Sapelovirus* and *Cardiovirus*, and the unrelated virus controls. This potential pan-PTV diagnostic reagent may be used to differentiate naturally infected animals from vaccinated animals that have antibodies against a subunit vaccine that does not contain this epitope or to screen for PTV before further subtyping. To our knowledge, this is the first report that utilized in silico PTV epitope prediction to find a reagent broadly reactive to various PTV serotypes.

## 1. Introduction

Since 1929, when the first outbreak of porcine teschovirus encephalomyelitis was reported in Czechoslovakia, this highly fatal disease, caused by the virulent *Porcine teschovirus* serotype 1 (PTV-1) Teschen strain had spread throughout Europe, causing significant economic losses. Over the last decade, other less virulent PTV-1 isolates were also causing epidemics worldwide [1], with or without complications caused by a variety of common swine pathogens, such as the post-weaning multi-systemic wasting syndrome-causing *Porcine circovirus type 2* (PCV2) [2], *Porcine respiratory and reproductive syndrome virus* (PRRSV), and the *Classic swine fever virus* (CSFV) [3,4]. Today, porcine teschoviruses (PTVs) are endemic in swine herds worldwide. Frequently, multiple serotypes of PTV co-circulate in an animal, herd, and area. Further complicating the situation is the fact that PTV currently has at least 13 different serotypes [3,5]. Previously, PTV serotypes 1–7 and *Porcine Sapelovirus* 1 (PSV-1) were considered to be strains of *Porcine enterovirus* (PEV) (Table 1). Then, 11 serotypes were recognized [6] before PTV-12 [5] and PTV-13 [7] were discovered. The multiplicity of PTV infections and multiplicity of PTV serotypes highlight the need for a teschovirus genus-specific reagent that is broadly reactive, to detect multiple PTV serotypes in one reaction, and yet, does not cross-react with other common picornavirus genera such as *Sapelovirus* and *Cardiovirus,* which are also common swine pathogens. Although a pan-PTV nucleic acid diagnostic reagent is available [8], to our knowledge, a pan-PTV serodiagnostic reagent is not.

In PTVs and other picornaviruses, the capsid protein VP1 is the major antigenic protein on the virion surface that contains the dominant neutralization epitopes [9]. The usefulness of VP1 in generating either a diagnostic reagent or a subunit vaccine was recently been highlighted [10]. Similarly, antigenic sites on the VP1 protein of PTV-1 are mainly localized in two regions, i.e., the GH loop (amino acids 183–195) and C terminus [11]. These regions are potential epitope candidates for developing a reagent for an immunological assay or a marker vaccine used against animal picornaviruses [12,13]. Recombinant epitope-based immunological assays have also been developed for the detection of many animal virus infections, including *Foot-and-mouth disease virus* (FMDV) [14], CSFV [15], PRRSV [16] and many others [10]. These assays, which are based on using recombinant proteins as immunogens, provide a simple, highly sensitive, non-infectious, and inexpensive tool for pathogen detection or serodiagnostic purposes. For PTV, the use of recombinant antigens to develop a pan-PTV detection assay has not been investigated yet.

Traditionally, antigenic site (epitope) discovery relies on epitope mapping using monoclonal antibodies. This approach is laborious, time-consuming, and expensive because of the need for pathogen cultivation and subsequent protein extraction. The prediction of linear epitopes from the protein sequences alone [17,18] or by using both the protein sequence data and three-dimensional (3D) structural data [19] was recently investigated. Such computer-aided predictions have reliably reduced the time and cost needed for the analysis of the immunodominant regions of proteins. 

Epitope-based peptide immunization in animals, however, has not been satisfactory because of the low immunogenicity of short, linear sequences (around 6–20 amino acids (a.a.) per sequence) of peptide epitopes. To improve immunogenicity, the peptide epitope is either conjugated to a carrier protein, is tandemly repeated [20,21], or is incorporated with an artificial universal T cell epitope, termed Pan DR Epitope (PADRE), together with an AKFVAAWTLK amino acid sequence [22], and a universal Tetanus toxoid B (Toxin B) epitope P2 (Q_830_YIKANSKFIGITEL_844_), a toxoid used to enhance cytotoxic T lymphocyte response [23]. This approach, to our knowledge, has not been attempted for PTV. 

This study aimed to create a serological reagent, with the aid of bioinformatics, that has broad reactivity to PTVs and yet can exclude the related genera of the family *Picornaviridae*.

## 2. Materials and Methods 

### 2.1. Propagation, Purification, and Inactivation of the Porcine Teschoviruses

SK-RST (ATCC CRL-2842) cells were infected with the reference PTVs (Table 1) at a multiplicity of infection (MOI) of 0.1. Once the infected cells showed cytopathic effects (CPE) of rounding, shining and floating, the supernatants were harvested and subjected to 3 freeze/thaw cycles. Cell debris was removed by centrifugation at 5000× *g* for 30 min. The virus was precipitated with 7% polyethylene glycol 8000 (PEG 8000) [24] and was purified by centrifugation through a 30% sucrose cushion at 30,000× *g* for 4 h. The final virus pellet was resuspended in 200 μL of PBS buffer solution. The 50% tissue culture infective dose (TCID_50_) was determined in SK-RST cells using the Reed-Muench method. The PTVs were chemically inactivated by incubating them with bromoethylimine (BEI) which was prepared as described by [25] at a final concentration of 3% for 24 h at 37 °C with gentle shaking. After incubation, residual BEI was hydrolyzed with 0.02 M sodium sulfite. Virus inactivation was confirmed by the absence of CPE when transfected into monolayers of SK-RST cells. The amount of inactivated virus was estimated from the viral titer of the sample prior to inactivation.

### 2.2. Bioinformatics Search for a Conserved Epitope on the Porcine Teschovirus Capsid Protein VP1

**Figure 1:**
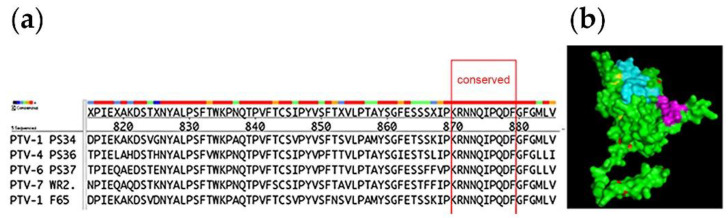
Bioinformatics prediction of an epitope conserved among porcine teschoviruses. (**a**) The left panel: Multiple sequences alignment of the GH loop (aa 183–195) of VP1 of 5 strains of PTV revealed the highly conserved “RNNQIPQDF” sequence. Note that amino acids 820 to 880 are numbered from the capsid polyprotein gene, which is constituted by VP4/VP2/VP3/VP1 genes. The polyprotein is later processed into individual proteins including VP1. (**b**) The right panel: The identified conserved RNNQIPQDF epitope (highlighted in purple) mapped within a 3D structure of the VP1 of PTV produced by the PyMOL visualization tool. The green area indicates the backbone of VP1 while the blue area indicates where the BC loop is located.

The following steps were employed to identify a conserved epitope on the VP1 of PTV. First, conserved peptides on the VP1 protein were identified by multiple sequence alignment [26] of the VP1 protein a.a. sequences of 5 strains of PTV (strains PTV-1 PS34, PTV-1 F65, PTV-4 PS36, PTV-6 PS37, PTV-7 WR2; Figure 1) retrieved from the NCBI GenBank database, performed using the ClustalW program available from the European Bioinformatics Institute (EBI) at http://www.ebi.ac.uk/clustalw. Amino acid sequences were taken from the UniProt Knowledgebase (http://www.uniprot.org/) and were aligned using the online multiple sequences alignment tool Clustal Omega [27]. Second, various in silico tools were used to predict the consensus immunogenicity [17], surface accessibility [28], and hydrophilicity of the conserved peptides identified [29]. Third, as the crystal structures and 3D models of the VP1 protein of the PTV isolates available in the protein data bank (PDB) were limited, it was assumed that the structure of the capsid proteins of the picornaviruses, such as *Mengovirus*, FMDV, *Human rhinovirus* and *Poliovirus* serotype 1 (PV-1), is similar. Therefore, the method used for the modelling of the PV-1 capsid protein (PDB code: 2PLV) described by [11] was used for the homology modelling (not alignment) and partial 3D structure modelling of the PTV’s loop structure, and to identify the ectodomains of the VP1 of the PTVs using the program Modeller 9.9 under default parameter conditions (http://www.salilab.org/modeller/9.9/release.html) [30]. The 3D structure modeling was carried out using the PyMOL software (www.pymol.org) to reveal the location of the conserved RNNQIPQDF sequence (the epitope) on the VP1 capsid protein. This conserved sequence was then subjected to immunogenicity and hydrophilicity analyses at a 1.000 threshold level. The homology model of the VP1 protein of PTVs derived using the PV-1 capsid protein model, was imported into PyMOL as the template. A surface model of the VP1 protein of PTVs was exported from the PyMOL software (www.pymol.org). For the antigenic sites to be accessible to the antibodies, it is most likely that these sites are located on the surface of a protein and that these surface regions are probably more hydrophilic than the interior regions. Algorithms for hydrophilicity and accessibility were used to predict antigenicity [29]. 

### 2.3. Construction and Expression of GST-Fusion Proteins Containing a Series of Linearly Arranged Epitopes (LAEs)

**Figure 2:**
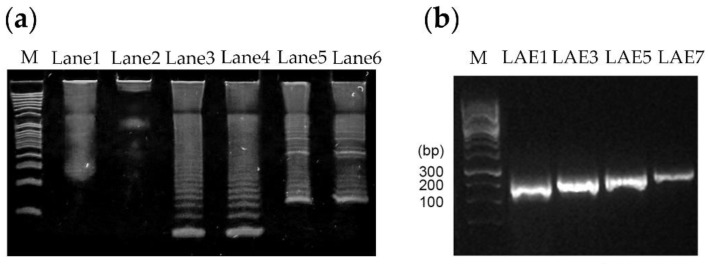
(**a**) PAGE analysis of the products generated using two different concentrations of oligonucleotide sets in the first (Lanes 1–2) and second rounds (Lanes 3–4) of TR-PCR, and adapter PCR (Lanes 5–6). Lanes 1 and 2: first round TR-PCR products using 100 pM and 10 μM of oligonucleotides, respectively. Lanes 3 and 4: corresponding products from the second round of TR-PCR. Lanes 5 and 6: corresponding products from the adapter PCR.; (**b**) PCR amplification of the LAE1, LAE3, LAE5 and LAE7 expression cassettes from the respective pLAE1, pLAE3, pLAE5 and pLAE7 pGEMT-Easy vectors showing product target sequences of 115 bp, 169 bp, 223 bp and 277 bp, respectively. PADRE primers were used for the PCR reactions.

**Figure 3:**
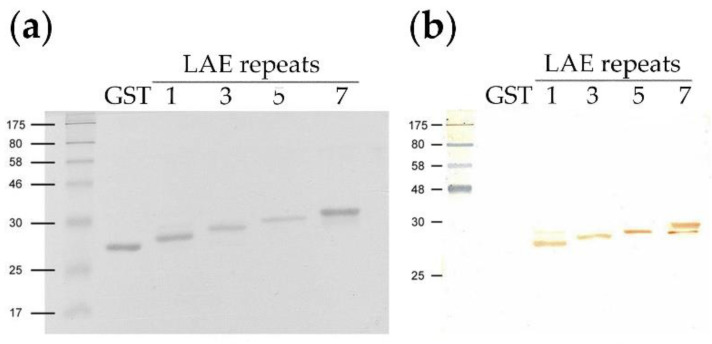
Western blot analysis of the gel-purified recombinant GST fusion protein (GST) and GST-PADRE-(RNNQIPQDF)n-Toxin B fusion proteins (LAE repeats: 1, 3, 5 and 7). (**a**) SDS-PAGE (16%) of the fusion proteins stained with Coomassie blue. (**b**) Western blot using the pre-pooled pig anti-PTV-1–7 serum as the primary antibody.

The coding sequence of the 9-a.a. long “RNNQIPQDF” VP1 epitope (oligonucleotide A, Table 2) was identified and its complementary sequence (oligonucleotide B, Table 2) was used as both the template and primer to generate a series of concatemeric linearly arranged epitopes (LAEs), by using a template-repeat PCR system (TR-PCR) [20], as explained in Table 2 and Figure 2. Two rounds of TR-PCR were performed to enrich the LAE amplicons, using two different dilutions of the oligonucleotide sets (100 pM or 10 μM) of the first round of TR-PCR; their respective amplicons were then diluted 100-fold for the second round of TR-PCR. The resulting amplicons from the secondary TR-PCR were used as the templates for adapter (overlapping) PCR (Table 2). The adapter-LAE amplicons obtained after adapter PCR contained various multiples of epitope coding sequences flanked by a HindIII restriction site with a PADRE sequence at the 5’ end, an XhoI restriction site with a Toxin B sequence at the 3’ end, and a stop codon at the end of the coding region (Table 2). These adapter-LAE amplicons were purified (nucleic acid purification kit (Favorgen Biotech Co., Pingtung, Taiwan) and to prevent too many tandem repeats from interfering with the exposure of the epitope to antibodies, amplicons smaller than 300 base pairs were cloned into the HindIII and XhoI restricted pGEM-T Easy vector as described in the manufacturer’s instructions (Promega Co., Madison, WI, USA). The resulting pGEM-T Easy clones, respectively carrying 1, 3, 5 and 7 LAE inserts were identified via sequencing (1st BASE DNA sequencing services, Singapore). These clones, designated pLAE1, pLAE3, pLAE5, and pLAE7, respectively, were digested using EcoRI and XhoI and were inserted into multiple cloning sites of the glutathione S transferase (GST) vector, pGEX 4T-3 (Amrad, Hawthorne, Australia). The resulting GST plasmids containing the respective LAEs were transformed into *Escherichia coli* BL21(DE3) and were grown at 37 °C for 4 h on a shaker (200 rpm) to express the GST-PADRE-(RNNQIPQDF)n-Toxin B fusion proteins (GST-LAE fusion proteins) after induction with 1 mM isopropyl-ß-D-thiogalactopyranoside (IPTG) for 4 h. A pGEX 4T-3 vector without an LAE insertion and expressing only the GST protein was transformed into DE3 (BL21) cell to obtain the GST protein, which was used as the control (Figure 3). 

### 2.4. Purification of GST-LAE Fusion Proteins

The GST-LAE fusion proteins (LAE1, LAE3, LAE5, LAE7) and the control GST protein expressed in bacteria were purified using glutathione Sepharose 4B columns (Cytiva, MA, USA) according to the manufacturer’s instructions as follows. The bacteria (25 mL) were centrifuged at 4000× *g* at 4 °C for 5 min, and the pellets were washed and suspended with 5 mL PBS. After another round of centrifugation and washing, the pellets were resuspended in 5 mL PBS containing 0.5 M benzamidine, 0.1 M phenylmethylsulphonyl fluoride (PMSF), 25 mg lysozyme, and 1% triton X-100 by shaking at 100 rpm at room temperature (RT) for 5 min. The bacterial suspensions were sonicated 6 times at 4 °C, each undergoing a 30-s sonication pulse followed by a 30-s rest (Misonix ultrasonic processor XL, NY, USA). The cell debris from the sonicated bacteria were pelleted by centrifugation at 18,500× g at 4 °C for 30 min, and the supernatants were harvested, filtered through 0.45 μm membranes, and loaded onto a PBS pre-equilibrated glutathione Sepharose 4B columns. The columns were first washed with PBS to remove low-affinity proteins, and the GST-LAE fusion proteins were then eluted with glutathione elution buffer (50 mM of reduced glutathione in 50 mM Tris, pH 8.0) at 1 mL buffer per mL bed volume. The eluted GST and GST-LAE fusion proteins were confirmed in 16% sodium dodecyl sulfate-polyacrylamide gel electrophoresis (SDS-PAGE) and stained with Coomassie Blue before being extracted via gel purification [31]. 

The eluted fusion proteins were purified from the polyacrylamide gel using a method modified from that of [31]. The distinct protein bands of the correct molecular weights of 27 to 30 kDa were cut out and washed three times in Eppendorf tubes with 250 mM Tris buffer/250 mM EDTA, pH 7.4, followed by rinsing 3 times with distilled water. The gel slices were then chopped to fine pieces before adding 20 mM Tris buffer, pH 7.4, containing 0.1% SDS (*w*/*v*) at a buffer to gel ratio of 2:1 (*v*/*v*), and were then sonicated (6 cycles of 30-s pulses and 30-s rest) in an ice bath using a high-intensity ultrasonic sonicator (Misonix, 50 Watts). After sonication, each of the gel slurries were spun down and the supernatant containing the GST-LAE fusion protein was harvested (Figure 3). The amounts of gel-purified LAE fusion proteins were quantified using the Bradford assay (Bio-Rad Laboratories, CA, USA). 

### 2.5. Mice Immunization and Production of LAE Antisera

The animal experimental protocols used were reviewed and approved by the Institutional Animal Care and Use Committee (IACUC) of Bestar Laboratories (S) Pte Ltd. where THT served. Each of the five groups of mice comprised of six 4-week-old female JCR mice were immunized with each of the respective gel-purified GST-LAE fusion proteins (LAE1, LAE3, LAE5 and LAE7) or the negative control GST protein. On day 0, each mouse was injected subcutaneously with 100 μL of antigen containing 25 μg of the GST-LAE fusion protein mixed with an equal volume of Freund’s incomplete adjuvant (Sigma -Aldrich, St. Louis, MO, USA). On days 14 and 28, the mice were bled and boosted with the same antigens mixed with an equal volume of Freund’s incomplete adjuvant (Sigma). An additional injection of 25 μg of protein antigen without adjuvant was administered at day 42 (without bleeding). Blood samples of 100 μL each were taken from every mouse by facial bleeding on days 14 and 28, and the mice were sacrificed after the final bleeding on day 45.

### 2.6. Western Blotting

Two primary antibodies were used in Western blotting to detect the viral proteins, listed as follows: (1) A pre-pooled anti-PTV 1-7 pig serum (1:200) (product code 363-PDV, National Veterinary Service Laboratory (NVSL), USA; www.aphis.usda.gov/animal_health/lab_info_services/downloads/AmesReagentManualCurrent.pdf); and (2) each of the GST-LAE fusion protein antisera produced in this study (1:500). The secondary antibodies were either horseradish peroxidase (HRP)-conjugated goat anti-mouse IgG antibodies (1:3000) (Dako North America, Inc., Carpinteria, CA, USA) or HRP-conjugated goat anti-pig IgG antibodies (1:3000) (Dako North America, Inc., CA, USA). The gel-purified GST-LAE fusion proteins and BEI-inactivated PTVs were electrophoresed in 16% SDS-PAGE and were transferred to nitrocellulose membranes for Western blotting as described by Liang et al. (2016) [32] (Figure 3). The protein bands were visualized by reacting with 3.3’-diaminobenzidine tetrahydrochloride (DAB).

### 2.7. Indirect ELISA for the Detection of the PTV VP1-Specific Antibodies in the Anti-LAE Antisera

**Figure 4:**
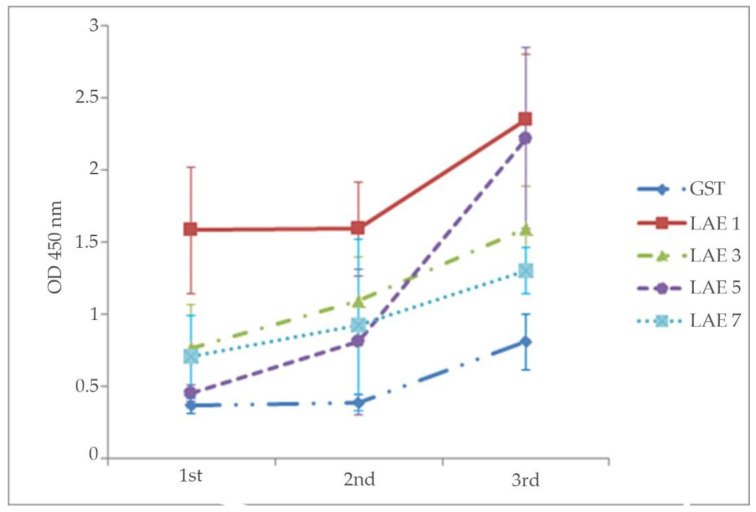
Measurement of rVP1-specific antibody activities in the antisera of mice immunized with the GST-LAE-fusion proteins (LAE1, LAE3, LAE5 and LAE7) by iELISA, using the rVP1 protein as the antigen. The GST-fusion protein antiserum (GST) was used as the negative control. The ordinate indicates absorbance (OD450 nm) of the antisera (abscissa) when reacted against rVP1. Note that there were 4 immunizations done at days 0, 14, 28, and 42; and there were 3 bleedings done at days 14, 28, and 45. 1st = 1st bleed at day 14; 2nd = 2nd bleed at day 28; 3rd = 3rd bleed at day 45. The data are presented as the mean absorbance ± 1 SD (n = sera from the 6 mice in each group).

The levels of anti-LAE immunoglobulins in the antisera were determined by indirect ELISA (iELISA) using a recombinant VP1 protein (rVP1) of PTV-1 PS34 prepared in our laboratory (Figure 4). Briefly, flat-bottomed microtiter plates were coated with 0.5 μg of rVP1 diluted in the standard coating buffer. Following standard blocking with 1% bovine serum albumin (BSA), a 1:1000 fold diluted 50 μL solution of each of the antisera was added to duplicate wells and incubated for 1.5 h at RT. After triple washing with PBS-T, 50 µL of the HRP-goat anti-mouse IgG antibody (1:3000; Dako North America , Inc., CA, USA) or HRP-goat anti pig IgG (1:3000; Abcam, Cambridge, UK) was added to each well. The reaction was developed by adding 100 μL of 3, 3’, 5, 5’-Tetramethylbenzidine (TMB) substrate per well and incubating the wells for 10 min in a dark room and then terminated by adding 50 μL of 2 M H_2_SO_4_. The optical densities (O.D.) of the reactions at 450 nm were determined with a microplate absorbance reader (Tecan Sunrise, Switzerland). The O.D. value of the negative control GST protein antiserum +1 standard deviation (SD) was used as the cut-off value.

### 2.8. Dot Blotting

**Figure 5:**
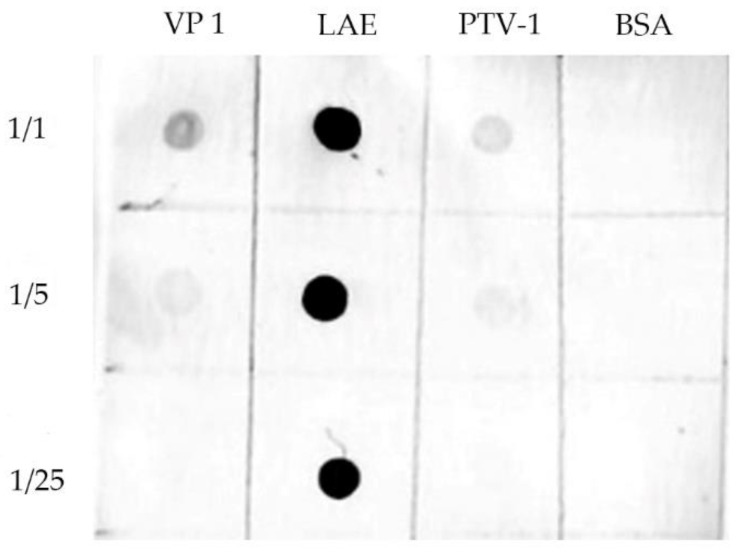
Assessment of the reactivity of the antiserum against LAE3 (LAE), and whole virions of PTV-1 PS34 (PTV-1) and rVP1 protein of PTV-1 PS34 (VP1) by dot blotting. BSA of similar concentrations started at 100 ng/µL served as an unrelated protein control.

Dot blotting (Figure 5) was conducted as described in the following protocol outlined on the Abcam website (www.abcam.com). Protein samples were concentrated to 100 ng/μL and were then diluted five-fold with PBS, while the inactivated PTV-1 PS34 virions (106 TCID50/μL before inactivation) were diluted with PBS. BSA used as an irrelevant control were diluted similarly, starting from 100 ng/μL. Nitrocellulose membrane blots were incubated with the antisera for 1 h at RT, followed by incubation with the secondary antibody alkaline phosphatase (AP)-conjugated goat anti-mouse IgG H & L (1:3000; Abcam, Cambridge, UK), and then with alkaline phosphatase chromogen (BCIP/NBT) (Abcam) for 10–30 min, for color development.

### 2.9. Immunofluorescence Assay (IFA)

IFA was conducted essentially according to the protocol outlined on the Abcam website (www.abcam.com). SK-RST cells with over 90% confluency were infected with the reference strains of PTVs (Table 1) at an MOI of 0.1 for 20 h. After fixing with methanol for 15 min at −20 °C, the cells were washed and then incubated for 1 h at RT with the antisera or mouse anti-PTV mAb 040/4B1 (1:1000) (a gift from Dr. M. Dauber, Friedrich-Loeffler Institute, Federal Research Institute for Animal Health, Insel Riems, Germany) in PBS plus 0.1% BSA. Finally, the cells were incubated with goat anti-mouse-FITC (1:5000; Abcam) in PBS for 30 min at RT. Fluorescence was examined by an inverted microscope (Olympus, Japan) and was documented using the Nikon ACT-1 software.

### 2.10. Virus Neutralization (VNT) Assay

The neutralizing activity of each of the antisera was assayed in duplicate wells via a microneutralization test (modified from OIE, 2018), using the pre-pooled anti-PTV-1-7 pig serum as a positive control. After incubation of the antisera at 56 °C for 30 min to inactivate the complement, a two-fold dilution series of each antiserum was prepared, and 25 μL of each dilution was mixed with 25 μL of a 100 TCID_50_ PTV-1 virus preparation; resulting solutions were then incubated at 37 °C for 2 h to neutralize the virus in separate tubes. Each of the virus neutralization mixtures were then transferred to duplicate wells of a 96-well plate, with each well containing a 90% confluent monolayer SK-RST cell in DMEM plus 5% FBS. The VNT titers of the antisera were determined after the cells were incubated for 5 days at 37 °C. The VNT titer of the antiserum from each of the 6 mice in each respective group, obtained at each bleed, were determined, and the highest group average VNT titer that completely inhibited virus growth was recorded. OIE manual recommends the seropositive VNT titer to be set at ratios higher than 1:8.

## 3. Results

### 3.1. Identification of the Conserved Epitope on VP1 of PTV

Alignment of the deduced amino acid sequences of the VP1 proteins of 5 strains of PTV, namely PTV-1 PS34, PTV-1 F65, PTV-4 PS36, PTV-6 PS37 and PTV-7 WR2, showed that the _869_RNNQIPQDF_877_ sequence was found to be 100% conserved among these viruses (Figure 1). The presence of this conserved sequence was also confirmed in an expanded alignment of the corresponding sequences of PTV-1 to PTV-13 (Table 3). PyMOL modeling revealed that the RNNQIPQDF sequence (the epitope) was located on the surface of a groove on the GH loop [11] of the VP1 capsid protein (Figure 1). Epitope prediction analysis showed that the epitope was highly antigenic and highly hydrophilic, indicating good surface accessibility. This highly conserved epitope was selected for further experiments in this study.

### 3.2. Construction of the Linearly Arranged Epitopes (LAEs) Expression Cassettes

Two rounds of TR-PCR reactions were needed to obtain the various PCR products that coded for different numbers of tandem repeats of the epitope RNNQIPQDF. Amplicons generated from the first round of TR-PCR, using 100 pM and 10 µM oligonucleotide sets, respectively, each showed a smear of stained substances of more than 300 bp (Figure 2a, Lanes 1 and 2, respectively). Although several discontinuous ladder patterns were observed, these were undefined. However, the products of the two second-round TR-PCR reactions showed well-distributed ladder patterns, which were indicative of a series of linearly arranged concatemers of the epitope coding sequence and an increasing number of epitope repeats (Figure 2a, Lanes 3 and 4, respectively). As the sizes of the products increased, the intensities of the DNA bands decreased. 

The products of the adapter PCR reactions (Figure 2a, Lanes 5–6) that introduced the restriction enzyme sites HindIII and XhoI, PADRE, Toxin B, and stop codon into the LAE constructs also showed a ladder pattern in the gel but were of higher molecular weights due to the adapter sequence. In lanes 3 to 6, the prominent lower molecular weight band in the ladders presumably corresponded to the monomer product of each reaction, while the higher bands corresponded to the trimer, tetramer, and so forth. Cloning of the adapter-LAE products into the pGEMT-Easy vector and sequence analysis of the resultant pGEMT-Easy-LAE clones confirmed that clones pLAE1, pLAE3, pLAE5, pLAE7, carrying the coding sequences of either 1, 3, 5 and 7 linearly arranged copies of the epitopes, respectively, were obtained. The LAE expression cassettes obtained from these clones and carrying the LAE1, LAE3, LAE5, LAE7 coding sequences with the expected respective sizes of 115 bp, 169 bp, 223 bp, and 277 bp (Figure 2b) were successfully cloned into the pGEX4T-3 vector, generating clones that expressed the corresponding GST-LAE fusion proteins in *E. coli* BL21 (DE3). 

### 3.3. Purification and Western Blot Analysis of the GST-LAE Fusion Proteins Containing the Linear Arrangement of Epitope (LAE) Antigens

The Sepharose column-eluted GST and GST-LAE fusion proteins (GST, LAE1, LAE3, LAE5, LAE7) produced distinct protein bands when analyzed via PAGE. Western blotting analysis of the gel-purified fusion proteins (LAE antigens) confirmed the presence of proteins of increasing sizes, with apparent molecular weights of 27 kDa (LAE1) to 30 kDa (LAE7), with the sizes of LAE3 and LAE5 in between (Figure 3a). A minor band was also detected in each of the LAE1 and LAE7 samples in both the stained gel as well as the Western blot. The one associated with the LAE7 antigen was of a lower molecular weight while the one associated with the LAE1 antigen was of a higher molecular weight. Western blotting of the GST-LAE fusion proteins with the pooled anti-PTV-1-7 pig serum demonstrated the antigenic activity for these proteins against the antiserum (Figure 3b). The minor bands observed in LAE1 and LAE7 also reacted with the anti-PTV 1-7 serum.

### 3.4. Production and Analysis of the Antibody Activities of the Anti-LAE Antisera against the rVP1 Protein

Antisera were harvested on days 14, 28 and 45 from the five groups of mice that were respectively immunized with the four LAE antigens and the GST control. At day 14, only the LAE1 antiserum had antibody activity significantly higher than that of the GST control; while by day 28, all the antisera had antibody activities significantly higher than that of the GST control. The antibody titers were the highest after the 4th immunization (3rd bleeding at day 45) with each of the LAEs, but without significant differences among those induced by LAE1, LAE3, and LAE5, as will be discussed later. The antibody activity in response to LAE5 was apparently delayed until day 45 when it was higher than those of LAE3 and LAE7 (Figure 4). The pre-pooled anti-PTV-1-7 pig serum positive control reacted with the rVP1 protein, validating the iELISA conducted. One-way analysis of variance (ANOVA; Microsoft Excel) on the antibody activities of the 3rd bleed antisera indicated a significant difference between the antiserum against GST and the LAE-antisera. There was no significant difference between the antisera against LAE1, LAE3 and LAE5. 

### 3.5. Evaluation of the Specificity of the Anti-LAE Antiserum against the GH Loop Domain by Dot Blotting, IFA and VNT

While the iELISA demonstrated the reactivity of the antisera with the expressed epitopes, it was also necessary to use dot blotting and IFA to test whether the antisera also reacted with the conformational form of the epitope. The dot blotting showed that the antiserum reacted strongly with the LAE3 antigen (Figure 5), and positively but weakly with the rVP1 and PTV-1 virions, probably due to the high antigenicity and immunogenicity of the PADRE and Toxin B proteins included in LAE3, which were not included in rVP1 and PTV-1. On the other hand, there were no cross-reactions observed with BSA. 

**Figure 6:**
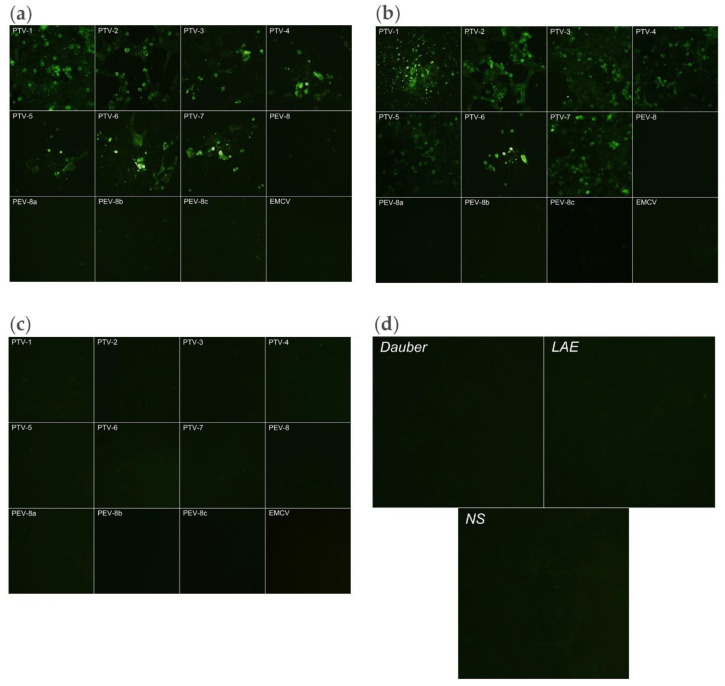
Reaction of the anti-LAE3 antiserum against PTV serotypes 1-7, *Sapelovirus* strains 436, and EMCV (a *Cardiovirus*) in IFA. (**a**) IFA reactions of the viruses tested using a pan-PTV mAb 040/4B1 mouse monoclonal antibody; (**b**) Corresponding IFA reactions using the anti-LAE3 antiserum; (**c**) Corresponding IFA reactions using a normal mouse serum and (**d**) IFA with antiserum to an unrelated (heterologous) mouse anti-enterovirus EV71 used as a negative control. The viruses tested was the PTV-1 PS34 strain (Table 1). Magnification: 200×.

The specificity of the antiserum was further demonstrated by IFA (Figure 6), which showed a broad reactivity of the antiserum against all the seven PTV serotypes (PTV1–7) tested, and no reactivity against the *Sapelovirus* strains and EMCV, a *Cardiovirus*. 

All LAE antisera showed no detectable virus neutralization activity at their original concentrations (i.e., no dilution or lower than 1:2 VNT). However, the pre-pooled anti-PTV-1-7 pig serum (used as the positive control) neutralized the PTV-1 protein at dilutions higher than 1/32.

## 4. Discussion

Although bioinformatics has been applied in a few other viral systems [13,32,33], to our knowledge, this is the first attempt, at the antibody level, to use bioinformatics in searching for and making antibody-inducing epitopes from PTV. Former PTV studies focused on molecular epidemiology, molecular methods for diagnosis, and RT-PCR for confirming infection. 

In this study, a key conserved antigenic site on VP1 was identified using bioinformatics, and the antibodies raised from the derived LAE antigens generated results that were partly consistent and partly different from those defined by [11,34]. Using traditional monoclonal antibody techniques, we identified a pan-PTV mAb clone 040/4B1, that was broadly reactive to all 11 PTV serotypes (i.e., pan-PTV) tested via IFA [34]. This antibody recognized a VP1 epitope that was discontinuous (conformational) and non-neutralizing. In direct immunohistochemistry, this 040/4B1 mAb did not produce a positive reaction on tissues from pigs naturally infected with PTVs, although infections were confirmed through RT-PCR [3]. Using a combination of genotyping and antibody technology, we identified a VP1 conformational epitope containing a stretch of 21 a.a. that interacted with the EF loop of VP2 and elicited antibodies capable of neutralizing PTV-1 [11]. 

In this study, a stretch of 9 a.a. on VP1 that was conserved among all 13 PTV serotypes (Figure 1; Table 3) was identified. The antisera raised reacted with PTV 1-7 but had undetectable neutralizing activity against the virus. The antisera did not react with members of the closely related genera *Sapelovirus* and *Cardiovirus* (Figure 6; Table 1) as well, which are commonly present in swine herds. Thus, the LAE antisera have the potential to serve as a pan-PTV diagnostic reagent in the field where the multiplicity of PTV serotypes and multiplicity of the PTV infection occur.

In the iELISA against rVP1 (Figure 4), the antisera were tested against the isolated viral protein. In dot blotting and IFA, the antisera were tested against the viral epitope closer to its native form in the viral and infected cells (Figure 5 and Figure 6). In its native form, the epitope (RNNQIPQDF) is hidden in the canyon [35], and the expressed recombinant proteins, such as VP1 and PTV virions should be less accessible to the antibody than the LAE3 antigen, as also shown in Figure 5. Since this species-specific antiserum did not neutralize PTV-1 infection, this putative universal epitope, by itself, may be insufficient to mediate virus aggregation.

Although bioinformatics requires less time to map key antigenic sites than conventional antibody technology [36], its limitation is that probably more than 90% of the conformational B cell epitopes, which are predominantly neutralizing, are difficult to predict solely with in silico analysis [19,37]. Despite this limitation, bioinformatics is accurate and powerful in locating common antigenic epitopes, whether they are linear or conformational, neutralizing or non-neutralizing, especially in viruses that include a multiplicity of serotypes/subtypes, such as PTV and the influenza viruses [32].

Serum neutralization of viruses is in general, a serotype-specific functional test and thus, should be stricter than iELISA and Western blotting, and should be mutually exclusive to genus- specificity (thus non-neutralizing or below the VNT detection limit of 1:2), as seen in the antiserum raised (Section 3.5). The 9 a.a. epitope identified in this study is part of the 21 a.a. neutralizing and conformational epitope in its natural form identified by Kaku [11] on PTV-1. In contrast to Kaku [11], whose 21 a.a. epitope was “a naturally conformational, neutralizing and PTV-1 type-specific epitope”, the tandem repeats of the 9 a.a. in this study appears to be “a presumably linear, non-neutralizing, and PTV genus-specific epitope”, although its actual protein folding patterns are unknown. The serotype-specificity and neutralization activity may be partially determined by a.a. sequences that are present on other proteins involved in capsid formation, such as the VP2 protein mentioned above, similar to the situation in FMDV [38]. 

The Western blot revealed one minor protein band in each of the purified LAE1 and LAE7 antigens (Figure 3). As both the minor and major protein bands reacted with the PTV antiserum with equal affinity, they are likely to be the results of complications occurring during protein expression in *Escherichia coli*, such as those that served to rid the cells of abnormal and misfolded proteins and to avail the cells of critical regulatory proteins [39]. 

Similar reasons may likely explain the discrepancy seen between the calculated molecular weights of LAE antigens (29, 31, 34 and 35 kDa for and LAE7 respectively) (Table 2) and the actual molecular weights of 27 kDa (LAE1) and 30 kDa (LAE7) (Figure 3). In addition, this discrepancy in molecular weights would probably be due to the unknown folding pattern of the GST-fused antigens. However, due to their similar ladder patterns seen in both nucleic acid (Figure 2) and protein (Figure 3a) gels, as well as their Western blot analyses (Figure 3b), their authenticity should be confirmed. 

All the LAE antigens induced antibodies that reacted to rVP1, suggesting that the poor antigenicity of epitope-based immunization seen in other systems [40] could be compensated by the incorporation of the T-helper epitope, PADRE [41], and the carrier protein toxin B epitope [23]. This also indicated that the incorporation of these domains was effective in generating a homogeneous immunogen with a high content of antigenic peptide at a lower cost, without the need for chemical conjugation. Unexpectedly, the LAE1 antigen containing a single copy of the conserved epitope appeared to be more effective in inducing antibody production in mice observed in all three bleeds, than those containing multiple copies (LAE3, 5, 7 antigens) (Figure 3 and Figure 4). In addition, antibody production, in response to LAE5, was below those in response to LAE3 and LAE7 until after the 4th immunization (3rd bleeding) (Figure 4), when it reached a level approaching that of LAE1. In contrast, the antibody production in response to LAE3 rose gradually. The LAE3 antiserum was selected for further studies because it only required 1–2 cycles of TR-PCR to generate 3 tandem repeats (Table 2) and thus, could easily be generated. In contrast, higher copies of linear epitopes (LAE5, LAE7) may interfere with optimal specific antibody production, probably due to the influence of protein folding, leading to variant antigenicity [42]. 

In summary, the use of bioinformatics to locate a conserved epitope among multiple serotypes/subtypes within a virus species described in the study is of particular advantage. The TR-PCR technique could be used to enhance the immunogenicity of the epitopes without the need for a DNA template. Moreover, the antiserum generated could have specificity properties similar to those of a monoclonal antibody [21] in that it has the potential to serve as a DIVA tool in differentiating infected from vaccinated animals, as in the FMDV marker vaccine [12].

## 5. Conclusions

We present herein an in silico method for predicting a universal epitope that can be constructed as a tandem repeat for expression in a PADRE-Toxin B cassette to produce a corresponding antigen that can be used for producing an antibody against an antigen of low antigenicity. This strategy has the potential to produce antibodies of good quality for species-specific diagnostic applications.

## Figures and Tables

**Table 1:** Reference *Porcine teschovirus* (PTV), *Porcine sapelovirus* (PSV) and EMCV serotypes and their respective strains used in this study.

**Virus**	**Serotype**	**Strain**	**Former Designation**	**Source**
*Teschovirus*	PTV-1	PS34	PEV ^1^-1	ATCC ^2^
*Teschovirus*	PTV-1	03B	PEV-2	NVSL ^3^
*Teschovirus*	PTV-1	PS14	PEV-3	NVSL
*Teschovirus*	PTV-1	PS36	PEV-4	NVSL
*Teschovirus*	PTV-1	F-12	PEV-5	NVSL
*Teschovirus*	PTV-1	PS37	PEV-6	NVSL
*Teschovirus*	PTV-1	WR2	PEV-7	NVSL
*Sapelovirus*	PSV-1	PS27	PEV-8	NVSL
*Sapelovirus*	PSV-1	PS32	PEV-8a	NVSL
*Sapelovirus*	PSV-1	ECPOI	PEV-8b	NVSL
*Sapelovirus*	PSV-1	PS30	PEV-8c	NVSL
*Cardiovirus*	EMCV-1	EMCV-1	EMCV ^4^	NVSL

^1^ Porcine enterovirus; ^2^ American Type Culture Collection; ^3^ National Veterinary Service Laboratories, USA; ^4^ *Encephalomyocarditis virus*.

**Table 2:** Strategies used in template repeat PCR and adapter PCR to construct a suite of linearly arranged PADRE-(RNNQIPQDF)n-Toxin B expression cassettes for cloning into the glutathione S transferase (GST) vector.

Template Repeat PCR		
Oligonucleotide A	A1	A2
= (A1 + A2)	5’-AGGAATAACCAGA	TACCGCAAGACTTC-3’
Oligonucleotide B	B2	B1
= (B2 + B1)	3’-ATGGCGTTCTGAAG	TCCTTATTGGTCT-5’
TR-PCR ^2^ 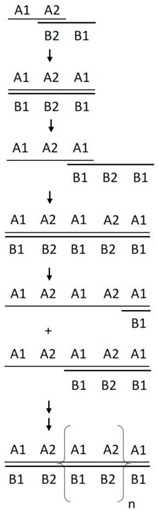	The oligonucleotide ^1^ A sequence (oligo A) encodes the target antigen (RNNQIPQDF) and the oligonucleotide B (oligo B) sequence is complementary to that of oligo A in the manner indicated above. Thus, the 5’ half of oligo A (A1) is complementary to the 5’ half of oligo B (B1), and the 3’ half of oligo A (A2) is complementary to the 3’ half of oligo B (B2). In TR-PCR, oligo A and oligo B were used as both primers and templates to generate multimers of tandem repeat epitopes. The cycling protocol comprised of 30 cycles of denaturation at 94 °C for 30 s, annealing at 37 °C for 30 s, and polymerization at 72 °C for 30 s, followed by a final polymerization step at 72 °C for 10 min. The product of the first round of TR-PCR is presented in lanes 1–2 of Figure 2, and that of the second round of TR-PCR is presented in lanes 3–4 of Figure 2.	
Anticipated genome arrangement after Adapter PCR		
Adapter PCR 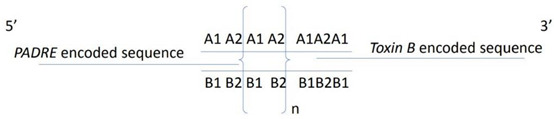		
Adapter primer A: 5’-GGTaagctt HindIII ATGGCCAAGTTCGTGGCCGCCTGGACACTGAAGAGGAATAACCAGATACCG-3’ Adapter primer B: 3’-TGAAGTCCTTATTGGTCTGTTATGTAGTTCCGGTTGTCGTTCAAGTAGCCTTATTGTCTCGAGATT XhoI gagctcCGG-5 The adapter primer A sequence contains the HindIII restriction site, the PADRE encoded sequence, and a part of oligo A to overlap with oligo B in PCR. The adapter primer B sequence contains part of oligo B to overlap with oligo A in PCR, the Toxin B encoded sequence and an XhoI restriction site. The underlined small letters indicate the HindIII and XhoI restriction sites. The underline capitalized letters indicate coding sequences of either the PADRE or Toxin B encoding sequences. The rest of the sequences are sequences of “oligo A1 and part of A2” to overlap with the sequence of “oligo B2 and part of B1” to adapt the PADRE and Toxin B sequences, as well as the restriction sites. The 100-fold diluted amplicon from the second round of TR-PCR (lanes 3–4, Figure 2) was subjected to adapter PCR with the adapter primers A and B. The amplification protocol was identical to that for TR-PCR, except that the polymerization step was performed for 1 min. The products of adapter PCR (lanes 5–6, Figure 2), which contained a HindIII site at the 5’ end, an XhoI site at the 3’ end, and a stop codon at the end of the coding region, were then subcloned into a pGEM-T easy vector.		

^1^ Oligonucleotide; ^2^ template repeat PCR. The amino acid sequence of PADRE is MAKFVAAWTLK; that of Toxin B is QYIKANSKFIGITE.

**Table 3:** Protein sequence alignment of the GH loops of the capsid proteins from the 13 serotypes of teschovirus.

Serotype	Strain	Alignment	End Sequence Number
PTV-1	PS34	LPAMYSGFETSSKIPKRNNQIPQDFGFGMLVLRSSFTA--GLAISV	896
PTV-2	T-80	LPTAYSGFETTFSIPKRNNQIPQDFGFGMLILRPSMPPTRKLVISA	899 ^1^
PTV-3	02b	LPTAYSGFETSYRIPKRNNQIPQDFGFGMLVLRSSSTL--GLAASV	897
PTV-4	PS36	LPTAYSGIESTSLIPKRNNQIPQDFGFGLLILRSSMPSPHELVASV	898
PTV-5	F26	LPTAYSGFESSSLIPKRNNQIPQDFGFGMLVLRSSSPT--ELVLSV	897
PTV-6	PS37	LPTAYSGFESSFFVPKRNNQIPQDFGFGLLVLRPSMPPTHKLVISV	899
PTV-7	F43	LPTAYSGFESTFSIPRRNNQIPQDFGFGMLVLRPSLPT--ELIISV	898
PTV-8	UKG/173/74	LPTFYSGIESTSLIPRRNNQIPQDFGFGMLVLRPSLSPANKIVVSV	899
PTV-9	Vir-2899/84	LPTAYSGFETSFSVPKRNNQIPQDFGFGMLVLRSSLQT--ELAMSV	895
PTV-10	Vir-461/88	LPVSYSGFETSYQVPKRSNQIPQDFGFGMLVLRSSSTT--KLTFSL	896
PTV-11	Dresden	LPTMYSGFESSSKIPKRNNQIPQDFGFGMLVLRSSSTL--GLTASV	897
PTV-12	CC25	LPTAYAGIESSSLIPKRNNQIPQDFGFGLLVLRSSMPPAHNLVVSV	899
PTV-13	JCAR2013SPN	LPTAYSGFETSNLIPKRNNQIPQDFGHGLLILRSSLRMDLVVSLWV	901

^1^ Note the amino acid sequences are numbered from the capsid polyprotein.
